# To adjust, reform, or transform? Criteria for distinguishing efforts to change knowledge practices in global health

**DOI:** 10.1080/17441692.2026.2707692

**Published:** 2026-07-23

**Authors:** Alice Bayingana, Seye Abimbola

**Affiliations:** a School of Public Health, Faculty of Medicine and Health, The University of Sydney, Sydney, NSW, Australia

**Keywords:** Knowledge practices, global health, changing practices, epistemic justice, distinguishing change

## Abstract

Over the last decade, there has been an increase in calls and efforts to change knowledge practices in global health, from within the field, in the fields that intersect with it, and from outside the field. While these calls and efforts are often aimed at rethinking or reimagining the field, they often fall short. Based on interviews with researchers who have participated in change efforts within and around global health, we developed criteria for distinguishing change efforts between first-order change, which seeks to adjust the current system for efficiency and effectiveness while keeping things the same; second-order change, which seeks to reform the current system to meet the needs of stakeholders; and third-order change, which seeks the total transformation of the system. In addition to the schema, our findings illustrate how the desired order of change is not simply a choice but rather dynamically determined in the interaction between change seekers and the institutions and systems they seek to change. This study offers a tool to clearly articulate the nature of a particular change effort, to distinguish and allow for comparison between change efforts, and to think through the kinds and extents of change required to achieve desired transformational change.

## Introduction

Calls for and efforts to change and reimagine global health knowledge practices—meaning practices involved in the production, use, and sharing of knowledge to advance health equity globally—have been ubiquitous for the past decade (Abimbola et al., 2024; Bayingana et al., 2025). From both within global health and in the fields and disciplines that surround and intersect it, criticisms and pushbacks have been articulated over the past three decades (Adams, 2016; Aellah et al., 2016; Bhakuni & Abimbola, 2021; Frankfurter et al., 2024; Naidu, 2021; Richardson, 2019; Storeng & Béhague, 2017). These have included concerns around the narrowing definition of evidence that has progressively excluded and rendered a lot of important knowledge and evidence untrustworthy (Feinstein & Horwitz, 1997). Others have criticised the oversimplification of complex phenomena and the abstraction of context in favour of generalisability (Marchal et al., 2013; Pawson, 1997, Pawson, 2004). Others have noted that problems and solutions which cannot be measured and evaluated in this way tend to be sidelined (Birn, 2009). Others yet have focused on the sidelining of values, preferences, and democratic deliberation, and the unaccountable power granted to expert research and policy technicians, often based in the global North over the lives of millions of people, who are mostly in the global South (Black, 2001; Drèze, 2020; Goodman, 1999). And others have complained about the sidelining of structural and social-historical understanding for an increasingly biological and behavioural frame (Birn et al., 2016; Navarro, 2008).

Following some scholars of global health and development, we trace the dominant knowledge practices that these complaints and change efforts problematise, which we refer to as evidence-based practice, to a set of developments that took place over the past 40 years (Birn, 2009; Donovan, 2018; Navarro, 2008). These developments are the reconfigurations of global health ushered in by neoliberal structural adjustment and the rise of evidence-based practice and policymaking with randomised controlled trials (RCT) as the gold standard of evidence. First, the rise of neoliberalism is understood to have instantiated a move away from the Health for All vision of the Alma Ata declaration of 1978 towards a shrunken and limited idea of what was possible policy and practice in global health (Birn et al., 2016; Birn, 2009). Through the defunding and dismantling of public health services and education, the privatisation of health and education, and a preference for market competition for the delivery of social services, the retreat of the state, and the rise of the NGO sector, funded by foreign aid agencies, international financial institutions, or new Private-Public Partnerships, along with the resurgence of behavioural and biological understanding of medicine and health (Birn et al., 2016; Laurell & Arellano, 1996). A new preference for short-term, measurable, cost-effective interventions over more structural and societal changes. Rights- and need-based arguments were considered wasteful and inefficient in favour of cost-effectiveness and value-for-money thinking (Birn, 2009).

Secondly, sharing concerns around wastefulness and inefficiency, there was spread of a particular idea of ’evidence’ to public health, global health, and beyond health into other arenas of social policy (Daly, 2005). This development is associated with Evidence-Based Medicine movement which popularised a pyramidal hierarchy of evidence on which clinical decisions should be made (Daly, 2005). This pyramid had RCTs at the top, as the gold standard of evidence. Methods of aggregating research using systematic reviews were developed, followed by guidelines from this aggregation of research that would then direct practice and policymaking. In the 2000s, a movement in development economics centred on impact evaluations via RCTs or modified RCT-like methods entrenched this even further in global and public health policymaking and practice, and beyond in other sectors of social policy and economic development (Birn, 2009). These developments have indelibly shaped the structure of knowledge practices that efforts today are trying to change.

Along with the critiques, alternatives, new and old, have been put forward for valuing the complexity and context of health and social interventions (Pawson, 1997), to centre the needs and preferences of people, service-users, and communities (qualitative, lived-experience, community engagement) (Greenhalgh et al., 2016), and to place biological and behavioural factors within a structural understanding of how health is determined (Krieger, 1994; 2011). There have also been efforts to challenge the view that health knowledge and innovations naturally flow from superior global North to the global South towards a view that this dynamic of technological dependency is structural and historical, and not natural (Fejerskov, 2017; Sekalala & Chatikobo, 2024). It is due to, and also a part of, centuries of underdevelopment in the peripheries and overdevelopment in the centres; extraction of resources, labour, and knowledge from the global South to serve the development of industry, scientific, and technological innovation in the global North (Fejerskov, 2017; Hickel et al., 2022, 2024). In a previous analysis, scholars sampled complaints and calls for change published in academic journals from 2017 to 2023 to construct a framework of expectations for dignity-based knowledge practices. In other words, starting from complaints about the ways in which the existing knowledge practices fell short of being able to deliver the goals of health equity and justice, they constructed a framework outlining expectations for knowledge practices that could deliver this goal (Bayingana et al., 2025). While critiques, calls for change, and alternatives have generated interesting discourse and experimentation at the margins of health research and have also forced certain adjustments to evidence-based practice, this has not been enough. Complaints and calls for change continue to echo longstanding critiques, even after many change efforts and reforms.

Embedded within calls for change and efforts to change knowledge practices in global health are ideas about how change happens, why it is needed, when needed, to what ends, and what the extent of the change is needed. Given the range of change efforts and the desires and expectations of the actors involved, a framework by which to make sense of the differences and similarities between them is useful. A set of criteria to distinguish these change efforts can help people understand and learn from the ways that others in and around the field think about changing knowledge practices. They can also help identify, clarify, and organise efforts, and help people assess and think through their own efforts and desires for change within the larger landscape. The aim of this paper is, therefore, to produce a framework that might help distinguish and describe the different ways in which change is conceptualised and approached in efforts to change knowledge practices in global health.

## Methods

### Theoretical framing

We approached this analysis with the goal of constructing a framework by refining an existing one, with the specific goal of distinguishing between different kinds of efforts to change knowledge practices in global health (including the applied fields that feed into it, especially public health, health policy, and medicine). For the construction of our own schema, we took inspiration from Pruitt and Waddell's schema (Table 1) for distinguishing orders of systems change, developed and described by in their 2005 working paper ‘*Dialogic Approaches to Global Challenges: Moving From ‘Dialogue Fatigue’ to Dialogic Change Processes*’ (Pruitt et al., 2005). Our construction was in part informed by previous adaptations and applications of the schema to analyse nutrition change strategies and policy proposals to change food systems (Lawrence et al., 2015; McLachlan & Garrett, 2008). These authors were interested in describing the extents and types of change that are implied or expressed in different kinds of policy efforts, that is, whether they aimed to adjust, reform, or to transform nutrition and food systems. They were also interested in trying to understand what kinds and extents of efforts could deliver the deep systemic change necessary to address the complex, social, and seemingly intractable problems the authors are concerned with: malnutrition and sustainable food supply (Lawrence et al., 2015; McLachlan & Garrett, 2008).

**Table 1.** Pruitt and Waddell’s criteria for distinguishing orders of change in problem solving initiatives.

Criterion	First order change	Second order change	Third order change
Desired Outcome	’More (or less) of the same.’	Reform	Transformation
Purpose	To improve the performance of the established system.	To change the system to address shortcomings and respond to the needs of stakeholders	To address problems from a whole-system perspective
Participation	Replicates the established decision-making group and power relationships	Brings relevant stakeholders into the problem-solving conversation in ways that enable them to influence the decision-making process	Creates a microcosm of the problem system, with all participants coming in on an equal footing as issue owners and decision makers
Process for change	Confirms existing rules. Preserves the established power structure and relationships among the actors in the system	Opens existing rules to revision. Suspends established power relationships; promotes authentic interactions; creates a space for genuine reform of the system	Opens issue to creation of entirely new ways of thinking about the issue. Promotes transformation of the relationships toward whole-system awareness and identity; promotes examination of the deep structures that sustain the system; creates a space for fundamental system change

To adapt this schema and as our starting points for distinguishing change efforts for knowledge practices in global health, we began with the two levels of demands—lower and higher—of the expectations for dignity-based knowledge practices in global health that were developed by analysing the complaints literature about knowledge practices in global health authored by health researchers (see Table 2). Building on the assumption that ends and means are not separable, we based our version of the orders of change on the spectrum of demands for dignity-based knowledge practices (Bayingana et al., 2025). In our construction of our schema, the higher demand of the expectations for dignity-based knowledge practices was taken to be the end goal of the transformative efforts to change global health knowledge practices. Following this, we reworked the second-order change in line with the ’lower demands’ of the expectations for dignity-based knowledge practices (see Table 2). The first-order change largely remained the same, and the existing rules in our schema referred to the rules for knowledge practices from the EBM. Once we had a provisional working schema derived as outlined above, we applied it to a sample of our data (see *Data sources* below) in order to refine it. During this process, we added ’problem framing’ as a criterion (alongside Pruitt and Waddell’s ‘purpose’, ‘participation’, and ‘process’). We found that identifying ‘problem framing’ was necessary because of instances in which it was from our interlocutors' ‘problem framing’ that what constituted ‘purpose’ for them could be inferred—while in other instances ‘problem framing’ could itself be inferred from how they articulated their purpose. Our interlocutors tended to describe the purpose of their efforts in terms of the problems that they sought to change. What constituted the content of the criteria of ‘purpose,’ ‘problem framing,’ ‘process,’ and ‘participation’ were informed by the expectations for dignity-based knowledge practices. These criteria were then refined during the interviews and using the original framework to analyse the interview data. This process produced a schema quite similar but also different from that of Pruitt and Waddell.

**Table 2.** Expectations for dignity-based knowledge practices split between lower and higher demand.

Expectation	Lower demand	Higher demand
Transparency	To acknowledge and account for different ways of making sense of what is being studied	To privilege the way that the researched know and make sense of what is being studied and provide justification for when it is not done
Democratisation	To give communities and participants in research a seat at the table on decisions in all steps of the process	To ensure communities and participants involved in research take the lead on all decisions in the research process
Non-extraction	To ensure that participants and researchers in their communities get material and learning benefits from being researched or participating in research	To ensure that the primary goal of research is to serve the material and learning needs of the people being researched
Transformation	To acknowledge and account for the structural and upstream conditions that determine the health of those being researched	To orient knowledge production towards supporting researched people and communities in their activities to understand and transform the structural and upstream conditions that determine their health

### Data sources

Our main source of data were qualitative interviews with researchers who had previously been or were currently involved in efforts to change knowledge practices in global health, public health, health policy, and medicine at the time of the interview. The interviews were conversational, guided by prompts, and informed by realist theory-building interview methods, wherein the interviewer could introduce their theoretical thinking in the interview. This means that the interviews served as data on the experiences of our interlocutors and as spaces of theorisation and insight generation because parts of our developing theories were presented to the interviewees who would challenge, build on, and respond to them. This means that during the interviews, our interlocutors were themselves participants in the theory-refining process.

Interview prompts were formulated by A.B. and S.A. Each interview lasted approximately an hour and was transcribed and de-identified for storage. Participants were recruited purposively based on A.B. and S.A.’s knowledge of the discursive and operational landscapes of efforts to change knowledge practices in global health based on previous work engaging in and analysing critiques of and efforts to change knowledge practices in global health (including in public health, health policy, and medicine) (Abimbola et al., 2024; Bayingana et al., 2025). Participants were also recruited through snowball sampling, where referrals from interviewees who met our criteria were followed up, and responders were invited to participate in interviews.

A.B. conducted the interviews, 41 were over zoom and four in-person. One interview began in person and, owing to scheduling constraints, finished over zoom. Forty-six participants were interviewed between October 2024 and November 2025. Of the 46 interlocutors, 27 were based in Europe and North America at the time of the interview (see Table 3). This is explained by the fact that throughout this project, global health is understood as efforts to advance health equity globally (Koplan et al., 2009). As such, our considerations for participant recruitment were not solely focused on the distance and power difference between the global North and South but also included gender, race, ethnicity, caste, disability, and class. Interviewees represented a wide range of areas of health research, including health systems, research ethics, maternal and child health, antimicrobial resistance, gender, race, migration, and health (see Table 3). At the time of the interviews, the interlocutors had previously been or were involved in various efforts to critique and change knowledge practices in global health and surrounding fields. (see Table 4). These efforts include public critiques (journal publications, participation in discourse online or at conferences); attempts to change particular knowledge practices of particular institutions (for example, curricula and community engagement practices); involvement in taskforces and working groups with the goal of changing practices; and involvement in programmes and projects that experiment with alternative knowledge practices. Change efforts that our interlocutors had previously been or were involved in at the time of interview ranged from those that called for or practiced epistemic pluralism, that is, those projects that challenge the epistemic narrowness of dominant global health knowledge practices, evidence-based practice, decolonising global health, equitable partnerships, those that advocated centreing on social determinants of health, and those that advocated for different forms of community participation.

**Table 3.** Summary of interviewee characteristics.

Characteristic	N
**Number of interviewees**	46
F	24
M	22
**Origin**	
North America	13
Europe	13
Asia	9
Africa	5
Australia	4
South America	1
**Current location**	
Europe	16
North America	11
Australia	7
Asia	6
Africa	5
South America	1
**Domain of global health work**	
Health Systems	10
Ethics	7
Development and health	6
Gender and health	6
Global mental health	5
Race and health	5
Public health emergencies	5
Education and training	4
Maternal and child health	4
Early childhood development	3
Family medicine/primary care	3
Indigenous health services	2
Health and human rights	2
Statistical methods	2
Sexual and reproductive health	2
Migration and health	2
Climate change	2
Implementation science	2
Preventive medicine	1
Occupational health	1
Digital health	1
One health	1
Rural health	1
Research publishing	1
Anti-microbial resistance	1

(Note: most interlocutors had more than one domain of global health work).

**Table 4.** Career stages, locations, and objectives of the change efforts of our interlocutors.

ID	Location	Career level	Objective of change efforts
I01	Australia	Early	Valuing lived experience; qualitative research, social theory, and indigenous and other marginalised world views in research, policy, practice
I02	Asia	Mid	Equitable partnerships, exploitation of global south researchers, decolonising global health
I03	Australia	Mid	Inclusion of indigenous people and perspectives in policy and practice
I04	Africa	Mid	Research integrity and fairness
I05	Europe	Early	Valuing qualitative research and the voices of marginalised people
I06	Europe	Late	Epistemic narrowness (valuing methodologies from the social sciences); linguistic pluralism (the sidelining of non-English speakers from global health discourse
I07	Asia	Mid	Epistemic injustice, decolonising global health
I08	Africa	Mid	Decolonising global health,
I09	Australia	Late	Race in health research and practice; health research with the goal of health justice and survival of indigenous people; indigenous health humanities
I10	Europe	Mid	lived experience in research, practice, and policy; global South as a site of knowledge production and theorisation
I11	*N*. America	Late	Critique of evidence-based medicine on values, ethics, uncertainty
I12	S. America	Mid	Community power, research towards health justice and health equity
I13	Europe	Early	Community perspectives and lived-experience in humanitarian emergency responses; decolonising global health
I14	*N*. America	Late	Racism and coloniality in health research, policy, practice; racism and coloniality impact on health outcomes
I15	Europe	Mid	Research for health equity, decolonising global health, decolonising global health education
I16	Australia	Late	Research and education for rural health systems
I17	Asia	Mid	Gender and class in health research, practice, policy; health research towards social justice
I18	Europe	Mid	Race in health research, practice, and policy; African health humanities
I19	*N*. America	Mid	Social sciences and humanities in health research; returning results to participants
I20	Europe	Mid	Social sciences and humanities in health research; social and cultural dimensions of health; race and gender in health research, practice, policy, decolonising global health
I21	*N*. America	Early	Critical theory in global health research
I22	Europe	Late	Equitable partnerships, transdisciplinary research
I23	Australia	Early	Equitable partnerships
I24	*N*. America	Late	Equitable partnerships, transdisciplinary research, community participation,
I25	Europe	Late	Community participation
I26	Africa	Late	Decolonising global health; equitable partnerships
I27	Europe	Mid	Social sciences and humanities in global health research; racism and coloniality as shapers of global health research and health outcomes
I28	Asia	Early	Equitable partnerships
I29	Europe	Mid	Social sciences and humanities in global health research and practice; epistemic exclusion or global south knowledge and critical knowledge from the global north
I30	Europe	Mid	Dignity and respect in development initiatives and research
I31	Africa	Mid	Equitable partnerships, equitable funding, decolonising global health
I32	Africa	Mid	Equitable partnerships; ethics of image use in global health
I33	*N*. America	Mid	Equitable partnerships, decolonising global health
I34	Asia	Mid	Decolonising global health
I35	*N*. America	Late	Ethics and values in medical and global health education
I36	Europe	Mid	Community participation
I37	Australia	Early	Lived-experience—valuing the views and preferences of health service users
I38	*N*. America	Early	Valuing qualitative evidence, evidence for social progress
I39	Europe	Late	Quality of statistical methodology in health research
I40	*N*. America	Late	Ethics in development research
I41	*N*. America	Late	Decision focused evaluations in development, quality evidence for social impact
I42	Asia	Late	Decision focused evaluations, quality evidence for social progress
I43	Europe	Retired	Quality of medical and health research
I44	*N*. America	Late	Quality of medical and health research, Critical appraisal of evidence
I45	Europe	Late	Social science approaches; more diverse evidence
I46	Australia	Late	Community participation

### Data analysis and synthesis

Following each interview, the interviewer took brief notes on the general narrative of the interlocutor's professional biography and any notable theoretical insights that may have presented themselves or were discussed during the interview. After the initial 13 interviews, transcripts were created and shared with the research team (A.B. and S.A.), and a series of analysis meetings were held where all reviewed the transcripts and discussed what was coming up and how to approach the analysis. After these meetings, minor adjustments were made to interview prompts. For example, a prompt was added to capture interlocutors’ reflections on the effect of the social context surrounding the mobilisation of change efforts, following some of our interlocutors mentioning that they joined efforts to change global health during the 2020 Black Lives Matter protests. Midway through the interviews, another series of analysis meetings were had where selected transcripts were shared and discussed in a series of meetings. After these meetings, the rest of the interviews were conducted, and the rest of the analysis was carried out by A.B., with frequent discussions and input from A.B.

The transcripts were read and coded using Microsoft Word. Extracted from the interview transcripts into Excel were characteristics (gender, origin, career level, current location), purpose of efforts, how the problem is framed, changes being sought, mentions of the change process, who is involved in the change, and how they are involved at what points. A separate word document was created that contained the summary narratives that started from the post-interview notes and were continually refined through transcript reading and analysis.

The interview data were analysed in three ways. First, the interviews were treated as professional biographical narratives of the interlocutors’ lives and journeys through health research. Second, they were treated as sites of co-constructed theorisation according to realist interviews. Finally, they were treated as sources of discrete answers to questions that could be aggregated and summarised. These three dimensions of interview data were used in the analysis. Some of the findings presented rely on the interpretation of interviews as professional narratives. Others rely on co-constructed theories. Others rely on the interpretation of a collection of answers to specific questions. In the findings section, where appropriate, relevant, and allowing for the maintenance of our interlocutors' anonymity, fuller quotes are added to provide examples.

As a descriptive analysis which sought to categorise inevitably normative change efforts, this analysis was, also inevitably, informed by the authors’ normative standpoint in relation to those change efforts. AB and SA are originally from Africa and were based in Australia throughout the duration of the study. Both have lived and been involved in academic, programmatic, and policy work in global health in both the global South and the global North. They have also been involved in calls for change and efforts to change knowledge practices in global health. This study is part of their ongoing work and commitments to addressing unfair knowledge practices and transforming global health research towards dignity-based knowledge practices (Abimbola et al., 2024; Bayingana et al., 2025). These experiences and commitments informed the authors’ approach to this analysis.

### Ethics

This study was approved by the Human Research Ethics Committee (HREC) of The University of Sydney [2024/143] in accordance with the National Statement on Ethical Conduct in Human Research (2007). Participation in the study was entirely voluntary and was based upon the participant signing a written informed consent form after receiving an information sheet with details about the study and their involvement and an opportunity to ask clarifying questions. In line with the terms of consent to which participants agreed, the data for this study are not publicly available, and all participants were de-identified by potentially removing identifying information and reporting findings as aggregated responses.

## Findings

Table 5 presents our schema for distinguishing between efforts to change knowledge practices in global health. The columns in the table represent the three orders of change: first, second, and third. The rows detail the four criteria for distinguishing change efforts or projects: what is the purpose or objective of desired change, how are the problems to be addressed framed, the process of change, who will be participating, and how will they be involved? The criteria are not entirely separable and are derived from explicit answers as well as inferred from narratives. Purposes and problem framing capture the general aims and goals of the change efforts or the interlocutor. Process captures how change efforts or people think about arriving at goals and aims. Participation refers to who is involved in this process, how they are involved, and the relationships between them. In our determination of the order of change in a particular change effort or way of thinking about change qualified for, where there was discrepancy across criteria, we prioritised process and participation. For example, change efforts may describe their purpose and the problems they seek to address in second order, but if their approach to process and participation meets the criteria for first-order change, this effort was considered to meet the criteria for first-order change.

**Table 5.** Criteria for distinguishing orders of change in efforts to change knowledge practices in global health.

Criterion	First-order change (Adjust)	Second-order change (Reform)	Third-order change (Transform)
Purpose or objective	To improve the performance of the established system.	To change the system to address shortcomings and respond to the needs of stakeholders.	To address problems of global health knowledge practices understood as part of the transformation of a larger unjust social, political, economic system.
Problem framing	The problem is a technical issue that is part of the kinks that have to be ironed out and is already being worked on	The knowledge system has glaring historical problems that can be fixed by reform programmes targeted to make up for the historical short comings but weighed against maintaining the functioning of the system.	The problem is bigger than just the knowledge system and it is fundamentally a social system designed to perpetuate the problems that we seek to study and solve as well as the problems of how we study and solve them.
Process for change	Through gradual self-correction: problems are identified in the normal working of the system, and solutions are created for the problems and the institution improved. Preserves the existing power structures and relationships between actors.	By challenging the unfair power dynamics shaping relationships within the system and their manifestations. Promotes engagement with a diverse range of actors.	By promoting structural and historical understanding and analysis of the problems of knowledge production, the problems of health inequality, and action to transform the whole system. Recognises and incorporates antagonism within the status quo and antagonism within change efforts.
Participation	Elite trained technical and methodological experts and funders.	Cross sectoral and cross disciplinary coalitions of expert professionals, and trans disciplinary engagement with representatives of communities and/or marginalised groups.	Actors with particular commitment to using knowledge to change current structures and relationships towards those that allow for more awareness, identification, and action to transform the whole system.

### First order change

The criteria for first-order change include efforts whose purpose was to adjust marginal technical inefficiencies that may have been overlooked before or newly arose. In this order, the major problems had already been addressed by standards and guidelines, leaving only minor technical inefficiencies, oversights, and mistakes to be quickly corrected so that the system can continue to operate at optimum efficiency. The process of change in this order reflects the current status quo’s theory of how change occurs: high-quality evidence is produced on a suspected problem and/or solution to the problem, which is then used to inform decision- and policy-making on a continuous basis. This can be generally described as gradual self-correction, in which all the major problems have been accounted for, leaving only system updates, finetuning, and maintenance as we learn more and notice mistakes in need of correction. Participation is largely relegated to specially trained technical and methodological experts whose job is to do this knowledge production and the creation of new methods, appraisal tools, guidelines, and best practices that everyone else then follows.

Many of the change efforts in our interviews that met the criteria of the first order were concerned with the quality of evidence and research. The purposes were largely to improve the quality of the evidence on which decisions for policy and programme implementation should be based, and problems were framed as problems of quality improvement for decision-making. In these frames, the process of change involved the elaboration of new and the improvement of old standards and guidelines, as well as the enforcement of existing rules and standards governing the production, appraisal, and use of evidence. The reasoning being that on matters of (producing and using knowledge to address the problem of) health inequality, ’The science is still evolving’ (I44), as one of our interlocutors put it. Participation was largely limited to specially trained technical and methodological experts who would carry out this setting of quality control standards that would then govern funding and research practice.

Another set of efforts that met these criteria was concerned primarily with the usability of evidence and research for decision making. Their main purpose was to adjust the generation of evidence and research to better serve the needs of decision makers. The problems in this set were framed around the notion that, as one of our interlocutors put it, ’academic incentives are not always well aligned with the needs of decision makers.’ (I42) The process of change involves a similar process of gradual improvement in which new problems and needs arise, are identified, and then met with solutions. Participation in this particular group of change efforts involves elite trained technical and methodological experts, the researchers they employ, and the stakeholders for whom they are producing evidence and knowledge, with the technical experts taking the lead on matters of knowledge production and aiming to serve the knowledge needs of the stakeholders.

Efforts were also considered to be first-order if their purpose and problem framing met the criteria for either the second or third orders of change, but the process and participation fell under first-order criteria. An example of this group of efforts are those concerned with the exclusion of particular marginalised groups from the research process and from the benefits of research that describes their problem as historical exclusion and their purpose as one of addressing the shortcomings that result from such exclusion, but whose practices and participation involve advocating for more training of members of this marginalised group to join the ranks of the technical and methodological experts involved in the creation of standards and guidelines. This was also the case for some efforts concerned with the exclusion of certain kinds of knowledge from the consideration of what counts as quality evidence, whose purpose and problem are framed in the second order but whose process and participation are in the first order. These include seeking to address this exclusion by advocating for technical and methodological experts to devise standards of quality and guidelines to govern these sidelined forms of knowledge and knowledge production so that they may be raised to the standard of high quality that is determined by proximity to RCTs and in turn become included in the ’evidence base.’

Some second- or third-order change efforts can be gradually transformed into efforts that meet the criteria for first-order change after they have been successfully institutionalised. For this set of efforts, the purposes and problems were still described in terms of addressing the historical shortcomings of knowledge practices or transforming knowledge practices as part of a larger project to transform wider social, political, and economic relations. Participation and process, however, became focused largely on keeping institutionalised projects going and funded in an environment that is increasingly hostile to change. This was described as becoming stuck in a loop of producing more and more evidence and reports that describe the same problems and propose the same changes and/or producing more and more alternative guidelines for better knowledge practices with no real power to implement or enforce them.

### Second order change

The criteria for second-order change include descriptions of the purpose of the efforts as addressing particular shortcomings or oversights of a flawed but otherwise largely functional system of knowledge production and a set of practices. Problems in this order were often framed as shortcomings that needed to be addressed so that the system could better meet the needs of all stakeholders. At times, these shortcomings were understood as historical and/or structural but not always. Processes of change and participation in this order generally promote bringing together a diverse range of stakeholders with different levels of power as participants in coalitions (researchers, funders, representatives of communities of marginalised people, policy makers, etc.), drawing attention to how the current practices are unfair, unjust, or inadequate, and devising ways to address these problems within the frameworks of existing institutions, with an emphasis on dialogue, consensus, and the mutual benefit of all stakeholders.

Most of our interlocutors were involved in change efforts, which we classified as second order. This is not surprising, as this kind of thinking may be seen as the standard way of thinking about and approaching the need for large-scale social and policy changes that people encounter in their training, in the literature, and in their work. The change efforts that met all the criteria for second-order change were primarily concerned with diversity, inclusion, and the extractive nature of research. For some of these efforts, the exclusion of marginalised voices and perspectives was a large part of how the problems were framed. Problems were also framed as focusing on the exclusion of marginalised people from the decision-making processes surrounding the making of knowledge and evidence about health. Thus, different kinds of diversity and inclusion were sought to remedy this issue, including epistemic and methodological pluralism, the expansion of existing understanding of knowledge and evidence to include various perspectives and voices outside of research and academia in the research process, inclusion of sidelined voices through community engagement, engagement of people with lived-experience, and service users. One of our interlocutors captured the general purpose of these projects as rethinking ’what is excellence, what is evidence, and who is an expert?’(I7).

Other efforts were described as seeking to address the extractive relationship between researchers with different levels of power and resources and between the researchers and those who are researched. This was to be addressed through equitable partnerships across groups with power differences, efforts to disseminate results back to the people and communities involved in the research, and/or efforts to ensure that the research is translated into policies, programmes, and products that benefit those who are researched. The processes of change and participation for all of these efforts were largely envisioned to include various forms of cross-sectoral or cross-disciplinary dialogue and trans-disciplinary collaboration involving representatives from different stakeholder groups with different levels of power envisioned to have common interests, forms of advocacy that involve producing knowledge to raise awareness of and apply pressure to powerful stakeholders who are believed to be best places to alter or provide resources for the alteration of unfair structures. This was largely imagined as taking place within the boundaries of what is acceptable in the existing institutions. As one of our interlocutors warns, ’Don't surprise your executives, don't…You can stimulate them to be broader, but antagonising them generally doesn't work.’ (I22).

Some efforts whose purpose and problems were framed in third-order terms were considered to fall under second-order change if their approach to process and participation met the criteria for the second order. This can happen for reasons such as the hegemonic nature of the second-order understanding of change processes mentioned above; concessions made in pursuit of or in the process of institutionalisation, securing or maintaining employment, and funding acquisition; the constraints of working within the confines of institutional boundaries that determine what is acceptable and possible; and the frustration of hope in the possibility of wider societal change, as described by our interlocutors. For some, this is a conscious decision made in a time when wider change does not seem possible, a sort of tactical retreat and a way to make some concessions in order to keep things going and reproduce a critical perspective into the next generation of researchers and practitioners, taking a kind of ’trojan horse approach’ (I25) as one of our interlocutors put it, or working as a ’double agent’ (I1)—working within the institution and against it as another interlocutor put it.

For other efforts, it is a gradual shift towards the second order, particularly if institutionalisation is part of the process. Owing to the need for the desired change to become financially viable once institutionalised, efforts can either pivot towards or expand to let in things that are ’more palatable to the moment’ (I25), which alters the project itself even as it might still leave pockets of resistance. Some of our interlocutors described the pressure to scale back to a more ’reasonable’ or pragmatic position in the face of a lack of institutional accommodation for the changes they wanted to make to their practice: ’I was like, let's just finish this project. Rather than trying to, like, you know, incorporate all these principles into it, because we'll be shooting ourselves in the foot. This is my pragmatism coming against my principles, right?’ (I33).

While not all who experienced this kind of shift had complicated thoughts about it, some expressed frustration and exhaustion stemming from shifts in the efforts they were involved in. One of our interlocutors described the effects of this kind of institutional pressure as follows: ’it started with one report that looked into racism at [institution], right? And, you know, so they organised that, and, then they had working groups of, like, how to then deal with the feedback, and then, well, what do you do? Well, you can't just implement, you can't just change what [institution] is, really. So, what do we do? Well, we create more working groups. Asking for more feedback. Just exhausting everybody, emotionally, mentally.’ (I20).

### Third order change

Change efforts were considered to be third- order if their purpose was described in terms of addressing the problems not as isolated problems of research and knowledge production but as symptoms of and part of efforts to transform the larger unjust global, social, political, and economic systems. The problems were framed as bigger than the system of knowledge production. Unfair knowledge practices are considered to be a result of this system, as well as a way that it maintains itself, not considered to be errors or shortcomings that should be alleviated, but the result of purposefully maintained structures to be transformed. Processes of change aim to promote structural and historical understanding of the problems of knowledge practices and health, and to promote understanding and action at different levels among more powerful researchers, researchers who are marginalised, activists, and participants in social movements for health justice and other forms of justice, and people and groups who are subjects or potential subjects of research in order to transform the whole system. Participation in this order promotes changes in the structure and relationships of the research process that mirror those of society towards those structures and relationships that allow for greater awareness of the system as a whole and one’s evolving position in it, identification of shared interests and potential points of solidarity and allyship, and collective action. Efforts in this order approach changing knowledge practices as likely to be antagonistic.

Because of the reality of the antagonism involved in this order of change, the hostility of institutions towards these kinds of efforts, and the general marginalisation of people who find them compelling, it is unlikely that efforts in this order can freely and openly exist within institutions of knowledge production for long. This was reflected in the interviews. In the previous two sections, we touched on how these efforts can be transformed when institutionalised into second- or first-order efforts. Even though only a few were actively involved, at that time, in third-order change efforts, some of our interlocutors were calling for, expressing the desire for, or actively thinking about building efforts to change knowledge practices along the lines of this order. Most of our interlocutors who were thinking along the lines of this order of change were early career researchers, many of whom were precariously employed or in the middle of making decisions about long-term employment. They were also grappling with what it would look like to organise such efforts and what it would take, given the hostility of institutions to this kind of change, as outlined in the second-order section above. Some of our interlocutors were even grappling with the implications that attempting or having attempted such changes would have on their careers, were still frustrated with the funding/institutionalisation trap that changed efforts they were previously involved in, and some were slowly developing their connections with activist and community organisations outside academia in preparation for organising such efforts in the future. A few of our interlocutors who were thinking along these third-order lines were considering leaving, on their own volition, and sometimes in anticipation of being eventually pushed out of health research, the particular institutions where they were employed, or academia in general.

Some of this thinking was in response to recent global events that were present within the interview space: the genocide in Gaza and the repression of efforts to hold institutions accountable for their complicity and silence in these atrocities, as well as the election of Donald Trump for a second term that began with massive changes in the structure and funding mechanisms of global and public health. Along the same lines of current global events reigniting things, a smaller group of people that aligned with this order were some senior researchers who were either involved in third-order change efforts, shifted to second- or first-order efforts for some time, and had shifted back to thinking in third-order terms, or who started out as part of second-order efforts but due to being frustrated in their efforts to change things or due to events in the political world had shifted to third-order approaches and thinking.

One of our interlocutors was part of a third-order effort that was institutionalised and remained in the third-order form. Unlike many similar projects that became institutionalised within the institutions they sought to challenge, this effort was intentionally built outside the larger institutions. It was challenging and with primary funding coming from outside of the institutions even as it maintained ties to the institution. As they described: ’...we built our own little home, our intellectual home, basically. And we, our PhD students, studied out of that home instead of at the university campus, and we took on work where we embraced being an enemy of the state and found a lot of joy in it like it was heartbreaking, like some of the piece of work that we have done. But there was a freedom in being able to do the work that we knew we could see a function in the work.’ (I9) They were able to do this by taking advantage of a moment when institutions and funders were facing widespread reckoning with racism, sexism, and persistent colonialism, which produced an expanded willingness to fund these kinds of projects. They also approached this with a sober awareness of the fact that this moment would come to pass: ’But I also know the season. Under the current leadership. This is the season. I'm gonna make the most of the season, while the sun is shining. And build it, put in place infrastructure that can withstand when the season changes.’ (I9). Another interlocutor described how such rollback of change efforts unfold: ’But then, you know, they make you keep fighting the same battles, because once you get to the point, they're like, oh, actually, we're taking away everything that we've given, so now you have to, you know, fight for the same… for the lecture for the students about the history. The fight we fought five years ago, well, whatever. It… yeah, so they just exhaust you in that way.’ (I20).

## Discussion

This study presents a schema for distinguishing between efforts to change knowledge practices in global health. Our findings also provide insight into how particular approaches to change are conceptualised, thought about, and pursued, and the contextual factors that might influence these processes. The schema presents three orders of change with four criteria: purpose, problem-framing, process, and participation. First order of change in which the desired outcome is more or less the same. No major change is required or desired. Change here is envisioned as regular adjustment to be carried out by specially trained technical and methodological experts to maintain the effectiveness and efficiency of the system as it is. The second order of change describes ’reform’ in which problems are understood as shortcomings of the system and, for the desired outcomes to be achieved, changes are acknowledged to be necessary to address these shortcomings and the needs of all stakeholders. Change here is envisioned as dialogue and building coalitions between all stakeholders to raise awareness about and challenge unfair dynamics. Finally, the ’transformative’ level, in which problems are not shortcomings, but rather constitutive features of the system. Desired outcomes require major changes that include but extend beyond knowledge practices to wider political, economic, and social systems. Both the status quo and process of change are understood to be antagonistic. Change then requires some form of struggle by actors committed to understanding the structure in order to change it. Practice is privileged over intent and, therefore, where there are discrepancies between criteria, process and participation are taken as the determinants of the level of change.

The schema that we refined and applied in this study has been previously employed as a conceptual tool to analyse efforts to change complex global challenges, such as efforts to transform food and nutrition systems (Lawrence et al., 2015; McLachlan & Garrett, 2008). Informed by the analysis of our participants’ experiences and the framework of expectations from previous research, the transformative process and participation were adjusted. While the previous applications of the schema take multi-stakeholder dialogue, collaboration, and coalitions as characteristics of transformative change efforts, our schema conceptualises this level of change as necessarily acknowledging and anticipating the antagonisms between stakeholders in the problem and between those involved efforts of change. This necessarily involves an element of struggle and resistance to change on the part of powerful stakeholders. This means that what is in the previous versions of the schema as ‘transform’ change process and participation is instead part of the ‘reform’ change process and participation in our schema.

To our knowledge, this particular schema has not been applied to global health as an object of or effort to change global health or its knowledge practices. However, our thinking about the variability within calls for and efforts to change knowledge practices in global health is in line with previous studies. Shawar et al present a spectrum of perspectives similar to the one we construct that ranges from reformist to transformative approaches (Shawar et al., 2023). In their schema, divergent perspectives in global health are connected to peoples' underlying ’neoliberal,’ ’democratic liberal,’ and ’neo-Marxist’ ideologies; with neoliberal and democratic liberal ideologies underlying reformist perspectives and the neo-Marxist ideology underlying the transformative perspective. The schema in this work is developed to understand how different actors in global health understand the concepts of ’resilience,’ ’self-reliance,’ and ’increasing country voice’ while ours is developed to understand how change is conceptualised by actors in efforts to change knowledge practices. For our purposes, a distinction between reform and an adjusted level was necessary to capture the important gradient within reformist approaches. In another schema, Wright describes three ‘modes of transformation’—symbiotic, interstitial, and ruptural—each with different strategies: symbiotic transformations aim to accumulate power within existing institutions to ultimately transform them, interstitial transformations build alternatives within the cracks of existing institutions outside of spaces dominated by people in power, and ruptural transformations seek direct confrontation with existing institutions (Wright, 2019). This schema has been employed to explore modes of degrowth, anti-capitalist, transformation, but has resonances with our schema (Chertkovskaya, 2022). All three of Wright's modes align with our schema's ‘transform’ third-order change, but symbiotic transformations may also align with ‘reform’ second-order change or initially appear as such, and as we found, may never proceed to ‘transform.’

Our findings hint at a dynamic process through which approaches to change are shaped by people responding to contexts, crises, circumstances, and the behaviour of institutions, which also have their own dynamic processes to deal with efforts to criticise and change them. Beyond political ideology that informs what kind and extent of change is desired and pursued, there seems to be an entrenched understanding of how change happens, as well as a general reactive approach to change. It is not to say that the kinds of political ideologies that Shawar et al. present do not play a part, but rather that ideology was one of many influences on the ways that people thought about change, and there was no consistency in ideological influence. In addition to the ideologies of those seeking to change things, our findings suggest that approaches are also shaped by exigent events outside of global health, such as resurgent social movements and events that destabilise the view of stability and progress, and by the conditioning effect exerted on people and projects seeking change by the field and institutions that they seek to change. Our findings also show that the reformist level of change was the most common way people thought about organising efforts to change knowledge practices, hinting at a sort of inherited or conditioned way of thinking about change from the field itself. According to Krugman’s in the analysis of the variations in understandings of ’decolonisation’ in global health, liberal reformism is ’the 'normal' standpoint, the easiest and most accepted way to imagine change’ in global health because the field is itself a liberal reformist approach to the problem of global health inequality and epidemic threats. This means that the dominant way that the field views change is as ’reformation or slight altering of (now global) structures and systems guided by Western-derived conceptualisations of freedom, democracy, and developmentalism’ (Krugman, 2023).

The interviews that were analysed in this research were conducted during a time of increasing uncertainty and change with regard to the future of global health research and the world system in general. Among the more salient changes were the publication by the United States government of a list of words that could no longer be used in funding applications and words according to which already allocated and potential future funding were withdrawn. These words include equity, gender, race, women, diversity, and inclusion. While these changes were not the subject of these interviews, they haunted the conversations that took place after January 2025. We recognise that the ground shifted significantly while we were in the middle of this project. Diversity, equity, and inclusion reforms have been greatly attacked (Amon, 2025). It is also uncertain what the doubling down on the EBM hierarchy of evidence—with renewed insistence that any issue that is not amenable to being studied using methods high on the hierarchy does not deserve to be studied, funded, or taken seriously—means for methodological accommodations and innovations that have so far been made to contend with the complexity of social reality and health as an object of research (Douthat & Boyd, 2026).

In this study, we aimed to distinguish between the different kinds of efforts to change global health knowledge practices and offer a tool that might aid in clarifying new efforts and analysing past efforts. These orders of change and the criteria for distinguishing them can help people involved in change efforts to articulate the dimensions of their desired change. This study adds to an ongoing discourse on power, equity, and justice in global health and its associated knowledge practices. It does so by bringing together the experiences of people involved in knowledge production and in the critical discourses and efforts to transform knowledge practices towards the goals of global health equity and justice beyond the global North/South divide to encompass other axes such as gender, caste, region, and ability. This study focuses on the general approaches to change. In a follow-up study, we will explore more closely the strategies and tactics employed in the change efforts our interlocutors were involved in and explore the dynamic processes and contextual and resource-related factors that influence thinking around choices of strategy and tactics. This study also lays the grounds for future work that may focus on further bringing these different people and efforts together to share knowledge and experiences and produce knowledge that supports their often-isolated efforts.

Calls for and organising towards changing global health have once again grown loud among many who have lost their livelihoods and those whose access to life-saving medicines and care has been affected by recent changes (Keynejad et al., 2026). In this moment, conceptual work that distinguishes between change efforts might spark new thinking about what kind and extent of change desired transformations in global health towards knowledge and associated practices that are well aligned with the aspiration for global health equity and justice will require.

## Conclusion

Global health and knowledge practices in the field have been the subject of many calls for change in recent years. Drawing on a previous conceptualisation of change efforts more broadly and of what constitutes desirable change, dignity-based knowledge practices in global health, and interviews with participants involved in previous and current change efforts in global health (including the applied fields that feed into it, especially public health, health policy, and medicine), this study constructs a schema for distinguishing efforts to change knowledge practices in global health. By doing so, we offer a tool that may help think through the practical considerations for new change efforts, articulate the dimensions of desired change, the differences and similarities among change efforts, strategies that may be more aligned to one order of change than another, and explain why some change efforts fail or succeed. However, we are aware that the proposed framework does not provide a solution. However, it is our hope that a better understanding of the kinds of and extents of change being pursued, as well as the kinds and extent of change required for transformation, will lead to better strategizing and planning in current and future efforts to change the field.

## Data Availability

Owing to confidentiality issues, interview transcripts cannot be made public. Deidentified excerpts from the interviews may be shared upon request.
